# Critical Consideration of Tuberculosis Management of Papua New Guinea Nationals and Cross-Border Health Issues in the Remote Torres Strait Islands, Australia

**DOI:** 10.3390/tropicalmed7090251

**Published:** 2022-09-19

**Authors:** J’Belle Foster, Diana Mendez, Ben J. Marais, Justin T. Denholm, Dunstan Peniyamina, Emma S. McBryde

**Affiliations:** 1 College of Medicine and Dentistry, James Cook University, Townsville, QLD 4811, Australia; 2 Australian Institute of Tropical Health and Medicine, James Cook University, Townsville, QLD 4811, Australia; 3 Torres and Cape Tuberculosis Control Unit, Thursday Island, QLD 4875, Australia; 4 WHO Collaborating Centre in Tuberculosis, Sydney Institute for Infectious Diseases and Biosecurity (Sydney ID), The University of Sydney, Westmead, NSW 2145, Australia; 5 Victorian Tuberculosis Program, The Royal Melbourne Hospital, Parkville, VIC 3050, Australia; 6 Department of Infectious Diseases, Peter Doherty Institution for Infection and Immunity, University of Melbourne, Melbourne, VIC 3004, Australia; 7 Tropical Public Health Services, Cairns, QLD 4870, Australia

**Keywords:** tuberculosis, Torres Strait, medical evacuation, cross-border

## Abstract

The international border between Australia and Papua New Guinea (PNG) serves as a gateway for the delivery of primary and tertiary healthcare for PNG patients presenting to Australian health facilities with presumptive tuberculosis (TB). An audit of all PNG nationals with presumptive TB who presented to clinics in the Torres Strait between 2016 and 2019 was conducted to evaluate outcomes for PNG patients and to consider the consistency and equity of decision-making regarding aeromedical evacuation. We also reviewed the current aeromedical retrieval policy and the outcomes of patients referred back to Daru General Hospital in PNG. During the study period, 213 PNG nationals presented with presumptive TB to primary health centres (PHC) in the Torres Strait. In total, 44 (21%) patients were medically evacuated to Australian hospitals; 26 met the evacuation criteria of whom 3 died, and 18 did not meet the criteria of whom 1 died. A further 22 patients who met the medical evacuation criteria into Australia were referred to Daru General Hospital of whom 2 died and 10 were lost to follow-up. The cross-border movement of people from PNG into Australia is associated with an emergent duty of care. Ongoing monitoring and evaluation of patient outcomes are necessary for transparency and justice.

## 1. Introduction

High tuberculosis (TB) rates in the Western Province of Papua New Guinea (PNG) (674/100,000 population in 2016) [1] and poor access to health services in the remote villages lead to many residents accessing health services at the Australia/PNG international border [2]. On Daru Island in the Western Province of PNG, an ongoing multidrug-resistant (MDR)-TB outbreak has been reported [3,4]. Cross-border movement of PNG residents of the Treaty villages places residents of the Torres Strait Islands, Australia at risk for TB and MDR-TB transmission [4,5], and options for critical healthcare needs are limited for PNG nationals living adjacent to the Australian border under current bilateral agreements [2,6].

Healthcare is available for residents of both Australia and PNG at primary health centres (PHC) located in the Torres Strait Islands, on the Australian side of the border. Furthermore, patients that present in a critical condition may be medically evacuated to an Australian hospital to receive advanced care [7]. In practice, health care delivery on the Australian side of the international border is supported by medical resources and access for Australian residents to health care services, medical interventions and follow-up [8]. The Australian Government has invested heavily in the TB control programme in the Western Province, particularly the South Fly region, however, health care provision remains inequitable in this region and location of residence has important implications for health outcomes [9].

### 1.1. Torres Strait/PNG Context

The Torres Strait Treaty is an agreement between the governments of both Australia and PNG, which was ratified in 1985 [10,11]. The Treaty provides protection for the local inhabitants and their traditional activities on both sides of the border and contains specifications for maritime jurisdiction, fisheries resources, and navigation [10]. Traditional inhabitants of 13 Australian Torres Strait Islands and 13 PNG Treaty villages enjoy cross- border movement without the need for a passport or visa, provided the intended travel is for traditional purposes [11]. The area that encompasses the Australian communities involved in the Torres Strait Treaty is known as the Torres Strait Protected Zone (TSPZ) (see map of Torres Strait/PNG border region at https://doi.org/10.6084/m9.figshare.16632823.v1, accessed on 14 January 2022; authored by J’Belle Foster, Marty Moran, Diana Mendez).

Health care is not considered a traditional activity, and as a result access to Australian health facilities for PNG nationals is not a provision under the Torres Strait Treaty [11]. However, residents from both Treaty and non-Treaty villages frequently visit primary health facilities located on Australian islands in the TSPZ. Queensland Health triages both Australian and PNG nationals, from Treaty and non-Treaty villages, who present to a PHC within the TSPZ according to the nature and immediacy of the clinical presentation [12,13]. It is a national requirement for Australian health services to have systems in place to recognise clinical deterioration [14], and in the Torres Strait early warning tool scores are routinely used, with greater flexibility for Australian residents when additional diagnostic workup or treatment is required [15]. The majority of PNG nationals who present to a health facility in the Torres Strait are not critically ill and are referred back to the PNG health system, provided they have a stabilised medical condition prior to discharge [12]. Queensland Health makes no provision for preventative or chronic disease care, however, patients who are critically ill cannot be sent back and require aeromedical evacuation [12].

A triaging service is provided by clinicians in the Torres Strait. Wound care and pain medications are provided by PHC clinicians and on discharge additional supplies are dispensed to the patient [8]. Although it is not within Queensland Health’s remit to invite PNG patients back for any follow-up care, laboratory results sharing, or treatment [13], in practice, frontline clinicians will often treat all PNG nationals, including repeated presentations. Anecdotally, this can include the administration of regular depot antipsychotics, which according to local policy would be considered a chronic condition for which treatment must not be provided [13]. It is unclear what the legal position is for limiting or denying health care from a human rights perspective given that the World Health Organization (WHO) International Health Regulations (2005) and United Nations Principles and Guidelines on Human Rights at International borders stipulate that health care may only be limited on significant public health grounds. As health care is not a provision of the Torres Strait Treaty, governance of health care at the border and associated moral responsibilities for PNG nationals and residents of the Torres Strait falls to Queensland Health and its frontline clinicians to navigate.

The Torres Strait is considered extremely remote by Australian standards and therefore does not have many of the health services afforded to residents of more populated areas [16]. Despite this, residents of the Torres Strait can access general outpatient care, with regular outreach clinics from visiting specialists and allied health services [17]. These services include vaccination, child and maternal health, sexual health, physiotherapy, mental health, and diabetes care [17]. Many PNG nationals living in the Western Province have limited access to health care infrastructure and have services that are often impacted by health worker shortages [18]. Inadequate healthcare availability is one of the reasons PNG nationals in villages closest to the Australian border choose to access Australian healthcare services in the TSPZ [18]. The distance from PNG to the Australian PHC on Boigu Island in the Torres Strait is 4.7 kilometres, which is easily accessible in a ‘dinghy’ (small motorised boat). By comparison, the closest PNG hospital is located on Daru Island, which is a 2–4 h boat journey for residents from the Treaty villages closest to Australia [18].

The cost to provide outpatient care, aeromedical transfer, and inpatient management of a critically ill PNG TB patient from the Torres Strait Islands to Australian tertiary health facilities was recently reported as $124,280 (Australian Dollars) [19]. While the financial cost of aeromedical services is heightened by remote health care requirements, there are personal stakes for patients who present severely unwell, and for clinicians who are required to make difficult decisions regarding optimal treatment for these patients balanced against relevant government policies. In the Torres Strait/PNG border region, clinicians are expected to function in a complex health system environment limited by the scope of practice, available clinical tools, and spoken and unspoken policy and funding constraints [20]. There is difficulty in getting the ‘balance’ right between allowing humanitarian healthcare access, while limiting excessive healthcare expenses and supporting services in PNG. Delivering health care within the politically defined boundaries of the TSPZ may present additional challenges which may influence clinical decision-making and the care provided [21].

### 1.2. Management of PNG Nationals with TB

TB is a disease with a protracted natural history, which presents a major public health challenge in PNG, with particular concern about the transmission of highly drug-resistant strains in the Western Province [4]. Managing PNG nationals with possible TB in the TSPZ poses major clinical, logistical, ethical, political, and financial challenges at the interface of both jurisdictions. Patients requiring ongoing management and care are generally referred to Daru General Hospital in the Western Province of PNG. From October 2020, an upgraded health centre equipped with X-ray facilities opened for PNG patients at Mabadauan, a Treaty village adjacent to the Australian border [22].

As stipulated in the local Cross-Border Policy and Procedure documents for use on the Australian side of the border [12,13,23], clinicians need to complete patient observations (respiration rate, heart rate, oxygen saturation, blood pressure, temperature, level of consciousness, pain, and level of distress in paediatric patients) and document these in the relevant observations charts. The observation charts are known as the Queensland Adult Deterioration Detection Score (Q-ADDS), Children’s Early Warning Tool (CEWT), and Queensland Maternity Early Warning Tool (QMEWT) [24]. There are four CEWTs and selection is dependent upon the age of the child presenting—<1 year, 1–4 years, 5–11 years and 12–17 years. Early warning tools are mandated in Queensland and are used to recognise and respond to clinical deterioration by tracking observations [25]. Each set of observations recorded is allocated a predetermined score on the chart, which allows clinicians to both predict/anticipate rapid deterioration and rapidly detect deterioration as it occurs, and identify the severity of illness. Based on these scores, the observation charts prompt the interventions required to manage each patient. Medical decisions are most often determined remotely by physicians (Rural Generalist Practitioners) based in Thursday Island Hospital. Where medical intervention fails to stabilise the patient and reduce the acuity of a PNG patient’s presentation to a health facility in the outer Torres Strait Islands, an early warning tool score of ≥5 constitutes the criterion met for medical evacuation to an Australian hospital (Appendix 1 of the Cross Border Procedure [13]).

For patients with suspected TB that meet the aeromedical retrieval and transfer criteria, a negative pressure isolation room in an Australian hospital must first be identified before aeromedical evacuation can be arranged [23]. Most PNG patients that are medically evacuated are admitted to Thursday Island Hospital followed by a transfer to Cairns Hospital on the Australian mainland, once treatment for TB has commenced and the risk of infectivity is reduced. For the most complex and critically unwell patients, Thursday Island Hospital is considered a staging area and transfer to Cairns or Townsville Hospitals should occur as soon as practicable [23].

Since the restructuring and strengthening of TB services located on Daru Island in PNG in 2012 [26], no evaluation has been done to explore access to TB care and outcomes achieved in PNG nationals presenting with presumptive TB to health services located in the Torres Strait Islands. This paper aims to provide an overview of the policy narrative at the Torres Strait/PNG border and examine the factors impacting patient care and outcomes. This paper will demonstrate how the tools available for clinical decision-making impact the clinical management of patients presenting with signs and symptoms of TB.

## 2. Methods

A retrospective audit of all PNG nationals who presented to Queensland Health facilities in the TSPZ with signs and symptoms of TB between 2016 and 2019, including those that were medically evacuated, was undertaken as part of this study. TB case notification data were obtained from Queensland Health’s Notifiable Conditions System. Additional data sources used were patient charts, observation charts, Best Practice software, and the Excel spreadsheets used by the Torres and Cape TB Control Unit to record each health facility presentation of symptomatic PNG patients. The Torres and Cape TB Control Unit spreadsheets contain outcome data for PNG nationals referred back to the PNG health system, courtesy of shared data during visits by the Torres and Cape TB Control Unit to the TB Programme at Daru General Hospital or via correspondence with the Queensland Health Cross Border Communication Officer.

The WHO weight-for-age charts were used to identify if children fell beneath the 3rd percentile for age and weight [27]. PNG patients may be discharged if they can *be ‘stabilised to the extent that no foreseeable deterioration will occur during return to the place of traditional inhabitation or to a health care facility outside of Australia’* and have a Q-ADDS/CEWT/QMEWT score ≤4 (Appendix 1 of the Cross-Border Procedure [13]). Therefore, the Torres and Cape Hospital and Health Service is obligated to ensure the patient receives care in an Australian hospital via aeromedical evacuation if the Q-ADDS/CEWT/QMEWT score is ≥5. Where early warning tool scores were not documented in observation charts or in Best Practice software, Q-ADDS, CEWT, and Q-MEWT, observation charts were used to manually calculate the score for each set of observations recorded.

**Definition** **1.** *‘≤4’ denotes patients that scored <5 on the Q-ADDS/CEWT/QMEWT which reflects local policy.*

**Definition** **2.** *Age categories depicted in the results section were selected to reflect age categories used in the Q-ADDS/CEWT/QMEWT (<1; 1–4 years; 5–11 years; 12–17 years; ≥18 years).*

Descriptive statistics were generated using IBM SPSS Statistics, version 25 (2019, Armonk, NY, USA) and StataCorp, version 13 (Stata Statistical Software: Release 13. College Station, TX: StataCorp LP.).

Existing clinical practice was benchmarked against local cross-border policy and procedures [12,13,23]. These are:

(1) Policy 0090-Papua New Guinea traditional inhabitants presenting to Queensland Health facilities within the Australian Islands of the Torres Strait Protected Zone;
(2) Procedure 1244-Management of Papua New Guinea traditional inhabitants presenting to Queensland Health facilities within the Australian islands of the Torres Strait Protected Zone;
(3) Procedure 0222-Management of Papua New Guinea Nationals accessing healthcare within the Australian Islands of the Torres Strait Protected Zone, presumed to have or diagnosed with Tuberculosis.

Patients were eligible if they presented to an Australian health facility in the TSPZ with suspected or confirmed TB, regardless of where or whether the diagnosis was confirmed [23].

Ethical approval was obtained from the Far North Queensland Human Research Ethics Committee (HREC/17/QCH/74-1157) and James Cook University (H7380). Patients were not involved in this study and a waiver of consent was granted by the Far North Queensland Human Research Ethics Committee (HREC/17/QCH/74-1157). Authorisation to use case notification data was granted under Public Health Act application QCH/36155-1157. The authors have conformed to the principles of the Declaration of Helsinki.

## 3. Results

Of 213 PNG nationals who presented to a PHC in the Torres Strait between 2016 and 2019 with signs and symptoms of TB, 44 (21%) were medically evacuated. Two PNG patients managed by Daru General Hospital were included in this audit because they initially presented to an Australian PHC with presumptive TB but were subsequently referred to Daru General Hospital by the Torres and Cape TB Control Unit. Another patient who presented with presumptive TB had been diagnosed with MDR-TB at Daru General Hospital prior to presenting to an Australian PHC. This patient was included in the audit because local procedures support the management of TB in patients with suspected or confirmed TB disease and the investigators decided to include known TB patients presenting emergently in the audit [23].

Of the 44 PNG patients with presumptive TB that were medically evacuated, 19 were diagnosed with TB. Table 1 shows that of 19 PNG patients diagnosed with TB that were medically evacuated, 10 had an initial score at the presentation of ≥5. Thirty-seven percent of TB cases medically evacuated were <18 years of age and of these; 57% fell beneath the third percentile for age and weight.

**Figure 1:**
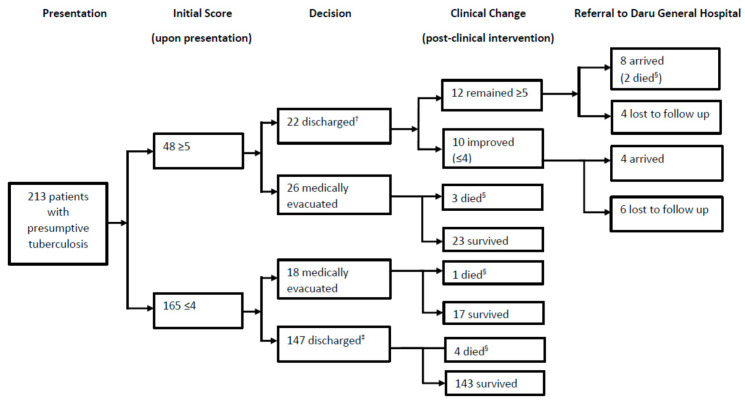
Outcomes of PNG nationals who presented with presumptive TB to primary health centres in the Torres Strait, Australia, between 2016 and 2019 according to their early warning score (Note. Initial score—this score is determined upon arrival to the health centre; ^†^ Includes four patients that did not have repeat vitals at time of discharge; ^‡^ Includes one patient that remained on oxygen at the time vital signs, including oxygen saturation levels, were being recorded prior to discharge; ^§^ It is unknown whether patients died due to tuberculosis or other causes).

Figure 1 shows the outcomes of all PNG nationals who presented with signs and symptoms of TB. Of this group, 10 (4.7%) died within the follow-up period. Of the 10 patients who died, six were diagnosed with TB, and of the TB patients who died three were not medically evacuated. Of the included patient cohort, 48 PNG patients had an early warning tool score of ≥5 upon arrival of whom 5 (10.4%) died. Those with a score ≥5 on arrival were 3.7 times more likely to die (95% CI 1.1–12) than those with a score ≤4 on arrival. Of the 48 high-risk patients, 26 (54%) were medically evacuated to an Australian hospital. Of the remaining 22 patients who initially presented with an early warning tool score ≥5 and were not medically evacuated, 12 were discharged with a score ≥5; 8 arrived at Daru General Hospital, and 2 of these patients died, with 4 lost to follow-up.

Of 80 PNG patients aged <18 years that presented to a PHC in the Torres Strait between 2016 and 2019 with signs and symptoms of TB, 13 (16.3%) patients had an initial score of ≥5, and of those, 8 were medically evacuated into the Australian health system. A total of 29 (36.3%) patients aged <18 years fell under the third percentile for age and weight and, of those, 10 were medically evacuated. Five patients aged <18 years who were medically evacuated with an early warning tool score ≤4 fell beneath the 3rd percentile for age and weight.

In undertaking the audit, the terminology ‘ceiling of care’ was repeatedly observed in the Queensland Health software, Best Practice. Typically, ‘ceiling of care’ describes a discussion that medical officers have with patients and their families in the context of futility of care for terminally ill patients. Hence, a separate data query on the term ‘ceiling of care’ was run. Terminology ‘ceiling of care’ was identified eight times in PNG patient’s Best Practice medical records, all of which were within this cohort and amounted to five PNG patients in total. A further PNG patient within the cohort had limitations on freedom of movement for healthcare applied but was not specifically labelled ‘ceiling of care’. Of the six patients with a ‘ceiling of care’ or similar restriction, the majority had chronic neurological problems, but none had known terminal conditions. Four of these six patients were children, aged 12 months–16 years.

## 4. Discussion

This study has identified inconsistent application of aeromedical retrieval policy, with patients not transferred for care despite meeting the criteria for medical severity and urgency. We report a range of poor outcomes in this cohort, including high mortality and loss to follow-up. Policy intended to avert such outcomes, particularly criteria for aeromedical retrieval to tertiary facilities, was not applied consistently. While our audit was not designed to explore the reasons for divergence from retrieval policy, our observations regarding the informal application of ‘ceiling of care’ without available documentation of reason for futility of care or case conference to discuss patient needs may indicate that such decisions are influenced by additional factors [28].

The pressures on clinicians to provide sound clinical judgment—often life and death decisions—while simultaneously avoiding costly care from patients who fall outside the Queensland Health remit, may lead to silent suffering and a risk burden on frontline clinicians that may be well beyond their training or experience [20].

There are many factors that lead to the high caseload in clinics in the Torres Strait Islands, Australia [2]. There is a significant discrepancy between healthcare services and outcomes on either side of the border of which Treaty villagers are well aware. Poverty and minimal preventative health activity in rural PNG leads to high rates of illness in residents of the Treaty villages. Travelling to Daru General Hospital for PNG Treaty villagers experiencing a health crisis or medical emergency can be prohibitive in terms of risk to life, personal safety, and personal costs [29].

Under the current system, once a decision on cross-border patient care has been made by the treating physician, there is no recourse to recall patients in need of further assessment or follow-up as per local policy 0900 and procedure 1244 [12,13]. Adding to the complexity of these care barriers are delays in transporting PNG patients from the Treaty villages to Daru General Hospital, with an average wait of 120 days between 2017 and 2018 [30]. Delays in transport for patients referred to Daru General Hospital pose an increased TB transmission and mortality risk in the region, while adding to Queensland Health expenses when patients represent.

Health service policy states that patients must be stabilised prior to discharge back to PNG and health care may only be withheld if treatment poses a substantial public health threat to community members (local policy 0900), in accordance with numerous statements on human rights [12,31,32]. As per local policy 0900, the costs of aeromedical evacuation and inpatient medical management should not guide clinical decision-making [12]. Despite this, medical decisions may be influenced by non-clinical factors, such as withholding care due to high cost, which has been reported in other settings [33,34]. This study highlighted that in the Torres Strait Islands, the phrase ‘ceiling of care’ may be used to mitigate other factors, such as cost or responsibility the health service takes for PNG nationals. As identified in the audit, in the Torres Strait, ‘ceiling of care’ is being used to indicate to other staff the limits that are to be placed on care such as fluid, inotropic agents or antibiotics, or restrictions of movement, such as the location in which the care stops. We did not investigate consent or whether decisions to limit care were unilateral as has occurred in other settings [35] but found no evidence in medical records of consent or multi-disciplinary conferences in decision-making.

PNG nationals who live across this border are ‘liminal’—existing in the space between—with both rights and restrictions placed on them by Australia [36]. There are, however universal human rights that apply regardless of political agreements, and it is contingent on these services to provide emergency care consistent with human rights. The liminal nature of PNG residents living adjacent to the Torres Strait Islands places a moral burden on healthcare workers to make determinations about the standard of care that will be provided. To avoid this burden being unreasonable, it must be consistent and transparent, both for the sake of PNG residents and for the healthcare decision-makers. We clearly risk harm to the PNG residents by denying needed care and by arbitrarily doing so. It may be less clear but also important that we also risk moral injury to decision-makers if we put them in a conflicted position regarding such choices, particularly where there are not transparent and objective criteria to guide them [37]. Monitoring and enforcement of standards is also a protection for healthcare decision-makers.

Early warning tools (Q-ADDS, CEWT and QMEWT) used in Queensland Health alert clinicians to vital signs of concern, using a colour-coded scoring system [25]. These tools are valuable in detecting severe bacterial sepsis or other emergent conditions likely to cause death; however, they were not developed to detect serious diseases relevant to the region including malnutrition and TB. In this study, 40% of patients that died having presented with signs and symptoms of TB were discharged back to PNG with an early warning Tool score ≤4. Conversely, 41% of patients who were medically evacuated had a score ≤4. Therefore, the scoring systems used may not be sufficiently sensitive to identify the most serious case presentations. For example, early warning tools do not allocate points to critical pathology results, and a patient with TB and pancytopenia may be discharged on a score ≤4 even though the patient may be experiencing a life-threatening medical emergency [38]. Further, the early warning tools do not allow for any allocation of points for failure to thrive or severe malnutrition, which are prominent features in paediatric patients with TB in PNG and can lead to rapid deterioration [39]. Using generic deterioration scores has been shown to decrease sensitivity to life-threatening conditions, particularly when used in specialty areas of care [40].

This study was unable to fully identify on which basis clinical decisions were made to either refer PNG patients presenting to Australian PHCs back to the PNG health system or to medically evacuate them to an Australian hospital. Further information about care provided to PNG patients, outcomes for those referred back to the PNG health system, and information on the cause of death for those patients who subsequently died is needed to identify the spectrum of patients’ outcomes following clinical decisions made at the time. Further research is warranted to better assist clinicians working in this complex context to optimise clinical decisions and patient outcomes.

Patient review is an essential element of the management of acute presentations to ensure patients can be safely discharged from care. Without the possibility of patient follow-up and with very low rates of post-mortem coroner referrals or referral pathways and feedback, some decisions are made without necessary oversight, transparency, or health system support.

Implementation of local policy and procedures pertaining to the management of PNG nationals presenting to health facilities in the Torres Strait were formulated, in part, to reduce ambiguity and to provide clarity for remote area clinicians on the appropriate management of patients. Aeromedical evacuations are necessary to provide equitable access to people with critical medical needs in remote settings, but this comes at substantial cost to the health system [41]. The very high costs of care are well known to clinicians who must make individual decisions under uncertainty, placing a burden on themselves and potentially leading to a reluctance to medically evacuate, consequently leading to reduced care in some cases, with cost to patients. In view of our findings, Box 1 summarises some key recommendations.

Box 1 **Key recommendations for improving care for patients with presumptive tuberculosis presenting to health facilities in the Torres Strait, Australia.**

1. Orientation and training of all staff is required to adequately address complex operational challenges associated with remote health care delivery, including ethical and medico-legal issues associated with time-critical health emergencies [42];
2. In addition to the identification of patient deterioration using current early warning tool scores, implementation of clinical algorithms that are appropriate for TB patients and malnourished children is warranted [43];
3. Exploring factors that influence both nurse’s and physician’s responses to patient deterioration is required, including how peer-modelling may improve health care delivery and adherence to policy [44,45];
4. Ongoing monitoring and evaluation to ensure transparency and justice is required [46]. Outcomes shared with local stakeholders will promote greater transparency of decision-making, with rapid identification of skills shortages and deviations from policy or policy limitations and with continuous service improvements led by frontline nurses and clinicians [46];
5. Care pathways that include documenting a set of vital signs just prior to discharge and with a medical review for Q-ADDS/CEWT/QMEWT scores ≥5 may improve patient outcomes and visibility of deviating vital signs [47]. An automated notification within the existing health system software may be beneficial to (a) prompt clinicians to collect and record vital signs at discharge and (b) reduce deviations from policy and procedures [48];
6. Greater transparency into how ‘ceiling of care’ decisions are made for cross border PNG patients seeking healthcare via the TSPZ is required.

Note. Ceiling of care—describes a discussion that medical officers have with patients and their families in the context of futility of care for terminally ill patients; however, in this study, ‘ceiling of care’ was applied to PNG patients who did not have known terminal conditions.

While TB causes a substantial number of deaths in PNG, determining the cause can be difficult due to concurrent health conditions and a lack of access to autopsy services [49]. In this study, the cause and the timing of death were unknown for some cases. In some instances, it is unknown if death occurred during or before treatment commencement. Further, the Q-ADDS/CEWT/QMEWT score was not available at the time of discharge for all patients; hence the last recorded set of vital signs were used in this study.

## 5. Conclusions

While more effective and efficient models of care are being developed in the Western Province in PNG, it is likely PNG nationals with presumptive TB will continue to present at Australian clinics in the Torres Strait. Risk scoring tools that are not appropriately contextualised may limit the accurate identification of serious cases requiring aeromedical evacuation. Tools that can perform both initial triage and identify subsequent deterioration in TB patients are required. Tools that incorporate a longer timespan for potential deterioration are needed in view of the high rates of loss to follow-up and slow arrival to Daru General Hospital. In the meantime, consistent use of the best available tools will reduce the burden of responsibility on frontline health workers involved in the remote management of these patients and support medical decision-making that is transparent and committed to equity.

## Figures and Tables

**Table 1:** Characteristics of Papua New Guinea nationals diagnosed with tuberculosis requiring aeromedical evacuation from the Torres Strait, Australia from 2016–2019 (*N* = 19).

Characteristic	Hospitals Providing Care				
	Thursday Island Hospital *N* = 4	Thursday Island and Cairns Hospitals *N* = 12	Thursday Island and Townsville Hospitals *N* = 2	Thursday Island, Cairns, and Townsville Hospitals *N* = 1	Total *n* (%) (*N* = 19) ^†^
**Early warning tool score ≥5**	1	7	1	1	10
**Age, median (IQR) ^‡^**					23 years (9–38)
**Age group**					
<1 year	0	1	1	0	2 (11)
1–4 years	0	1	0	0	1 (5)
5–11 years	1	2	0	0	3 (16)
12–17 years	0	1	0	0	1 (5)
18–29 years	0	4	1	0	5 (26)
30–44 years	3	0	0	1	4 (21)
≥45 years	0	3	0	0	3 (16)
**Sex**					
Female	1	6	1	1	9 (47)
Male	3	6	1	0	10 (53)
**Disease site**					
Extrapulmonary	0	2	1	1	4 (21)
Pulmonary	4	8	0	0	12 (63)
Both XPTB and PTB	0	2	1	0	3 (16)
**Drug resistance**					
Clinical Dx only ^§^	0	5	1	0	6 (32)
Fully drug susceptible	4	5	1	0	10 (53)
Multidrug-resistant	0	2	0	1	3 (16)

Note. XPTB—extrapulmonary tuberculosis; PTB—pulmonary tuberculosis; Dx—diagnosis; multidrug-resistant (TB which is resistant to both isoniazid and rifampicin). Note. Three patients with meningeal TB were managed at Townsville Hospital because it is the nearest facility with neurosurgery. ^†^ Represents 19 patients who were both diagnosed with tuberculosis and medically evacuated. ^‡^ Interquartile range. ^§^ No microbiological diagnosis.

## Data Availability

As notifiable disease data have been used in this study, public sharing of data is restricted due to confidentiality clauses. Access to data requires Human Research Ethics Committee, Public Health Act and Site-Specific Access approvals via Queensland Health.
